# The interplay of dopamine metabolism abnormalities and mitochondrial defects in the pathogenesis of schizophrenia

**DOI:** 10.1038/s41398-022-02233-0

**Published:** 2022-11-07

**Authors:** Haiyun Xu, Fan Yang

**Affiliations:** 1 School of Mental Health, Wenzhou Medical University, Wenzhou, China; 2 Zhejiang Provincial Clinical Research Center for Mental Illness, The Affiliated Kangning Hospital of Wenzhou Medical University, Wenzhou, China; 3 Mental Health Center, Shantou University Medical College, Shantou, China

**Keywords:** Schizophrenia, Molecular neuroscience

## Abstract

Dopamine (DA) is a major monoamine neurotransmitter in the brain and has essential roles in higher functions of the brain. Malfunctions of dopaminergic signaling have been implicated in various mental disorders such as addiction, attention deficit/hyperactivity disorder, Huntington’s disease, Parkinson’s disease (PD), and schizophrenia. The pathogenesis of PD and schizophrenia involves the interplay of mitochondrial defect and DA metabolism abnormalities. This article focuses on this issue in schizophrenia. It started with the introduction of metabolism, behavioral action, and physiology of DA, followed by reviewing evidence for malfunctions of dopaminergic signaling in patients with schizophrenia. Then it provided an overview of multiple facets of mitochondrial physiology before summarizing mitochondrial defects reported in clinical studies with schizophrenia patients. Finally, it discussed the interplay between DA metabolism abnormalities and mitochondrial defects and outlined some clinical studies showing effects of combination therapy of antipsychotics and antioxidants in treating patients with schizophrenia. The update and integration of these lines of information may advance our understanding of the etiology, pathogenesis, phenomenology, and treatment of schizophrenia.

## Introduction

Dopamine (DA) is a major monoamine neurotransmitter in the brain although a substantial part of the overall DA in the body is produced by mesenteric organs [1]. It engages in higher functions of the brain including spatial memory [2], motivation [3], arousal [4], reward and pleasure [5, 6], in addition to regulating motor neurons [7], lactation [8], sexual behavior [9], and nausea [10]. Considering these vital functions of DA, it is not surprising that malfunctions of dopaminergic signaling have been implicated in the pathogenesis of various mental disorders such as addiction, attention deficit/hyperactivity disorder (ADHD), Huntington’s disease (HD), Parkinson’s disease (PD), and schizophrenia [11].

The synthesis of DA happens in cytosol through the enzymatic reactions catalyzed by tyrosine hydroxylase (TH) and aromatic amino acid decarboxylase (AADC). The former enzyme converts tyrosine to dihydroxyl-phenylalanine (DOPA), which is further converted to DA via AADC catalyzation having pyridoxal phosphate as a cofactor [12]. The catabolism of DA, however, involves mitochondrion and results in the production of reactive oxygen species (ROS). Under physiological conditions, the rate of DA oxidation is slow, and the cellular antioxidant machinery can cope with the formation of highly reactive products from DA oxidation [13]. In a state of elevated DA or increased DA oxidation, however, higher levels of DA is toxic to mitochondria of neurons and glia cells [14]. On the other hand, mitochondrial defects may lead to DA elevation in the cytosol because of impaired capacity to degrade DA [15]. This interaction between DA and mitochondrion has essential roles in the pathogenesis of PD and schizophrenia [11].

In the pathogenesis of PD, DA oxidation-associated oxidative stress (OS) drives α-synuclein aggregation, the primary structural component of Lewy bodies and a pathological hallmark of PD [16]. In addition, DA adducts can modify α-synuclein and promote its aggregation [17]. On the other hand, mitochondrial OS leads to oxidized DA accumulation, ultimately resulting in reduced glucocerebrosidase enzymatic activity, lysosomal dysfunction and α-synuclein accumulation in brain neurons [18].

Different from PD in which mitochondrial dysfunction and DA-associated OS lead to dopaminergic neuron loss in substantia nigra as reviewed above, schizophrenia patients show no consistent neuropathology [19]. However, there is increasing experimental and clinical evidence for interactions between abnormal DA metabolism and mitochondrial defects in schizophrenia, which has more complex and diverse clinical manifestations in categories of positive symptoms, negative symptoms, and cognitive impairment. Positive symptoms include delusion, hallucination, disorganized speech and behavior. Negative symptoms refer to lack of social interest, anhedonia, and reduced initiative and energy. Cognitive impairment is characterized by a wide range of cognitive defects, which may precede the onset of psychosis for years and continue to worsen after a diagnosis of schizophrenia [20–24]. Of these clinical symptoms, positive symptoms are attributed to subcortical hyperdopaminergia whereas cortical hypodopaminergic may contribute to negative symptoms and cognitive impairment [23].

Instead of making a comprehensive comparison between PD and schizophrenia in their pathogenesis, this review article intended to discuss the interaction between abnormal DA metabolism and mitochondrial defects in cells and relate it to the treatment of schizophrenia. It started with the introduction of metabolism, behavioral action, and physiology of DA, followed by reviewing evidence for abnormal DA metabolism in patients with schizophrenia. Then it provided an overview of multiple facets of mitochondrial physiology before summarizing mitochondrial defects reported in clinical studies with schizophrenia patients. Finally, it discussed the interaction between abnormal DA metabolism and mitochondrial defects and outlined some clinical studies showing effects of combination therapy of antipsychotics and antioxidants in treating patients with schizophrenia. The update and integration of these lines of information may advance our understanding of the etiology, pathogenesis, phenomenology, and treatment of schizophrenia.

## Dopamine: metabolism, dopaminergic pathways, behavioral, and physiological functions

After the synthesis, DA is incorporated into synaptic vesicles by the vesicular monoamine transporter-2 (VMAT2) and stored in the vesicles in catecholaminergic neurons [25]. Following an action potential to a dopaminergic neuron, DA in synaptic vesicles is released into the synaptic cleft and binds to either postsynaptic or presynaptic DA receptors (DRs) or both. All DRs are metabotropic and subdivided into two major groups: D1-like receptors including D1 and D5, and D2-like receptors of D2, D3, and D4 [26]. Activation of DRs initiates second messengers, which trigger or block the activation of specific cell signaling pathways in post-synaptic neurons [27].

After a synaptic transmission, DA is taken up into the cytosol by either high-affinity DA transporters (DAT) or low-affinity plasma membrane monoamine transporters. The DA in the cytosol is then repackaged into synaptic vesicles by VMAT2 [28]. DA in the synaptic cleft is taken up by the surrounding astrocytes where DA is degraded into its metabolites through reactions catalyzed by monoamine oxidases (MAOs) and catecholamine-O-methyltransferase (COMT) [29].

**Fig. 1:**
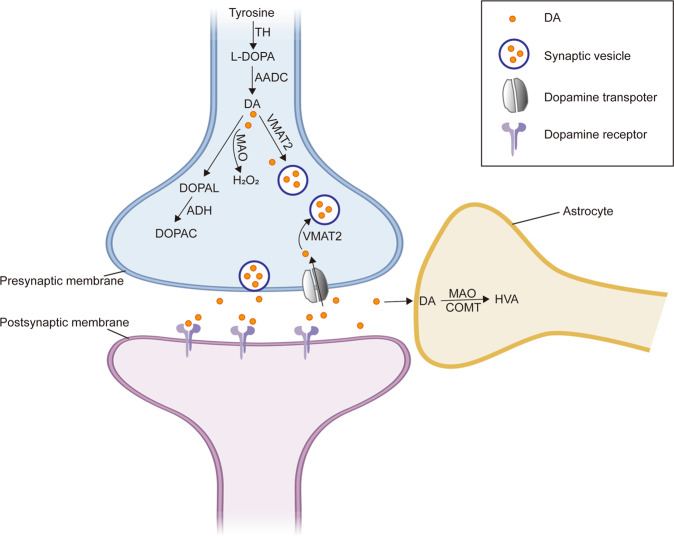
DA metabolism in dopaminergic neurons. In addition to the uptake of DA by the DA transporter (DAT) from outside, Dopaminergic neurons produce DA under the action of tyrosine hydroxylase (TH) and aromatic amino acid decarboxylase (AADC). The newly synthesized or taken up DA is stored in vesicles with the aid of vesicular monoaminergic transporter-2 (VAMT2). Cytosolic DA can be degraded in neurons or glial cells by catechol-o-methyltransferase (COMT) or monoamine oxidase (MAO) to form homovanillic acid (HVA) or be oxidized to form the metabolites (DOPAL, and DOPAC) and hydrogen peroxide (H_2_O_2_). The fig was modified from that by Zhang et al. (2019)^153^.

The degradation of DA to its inactive metabolites is carried out by COMT, monoamine oxidase A (MAO-A), or MAO-B. MAO breaks down DA to 3,4-dihydroxy-phenylacetaldehyde (DOPAL) and hydrogen peroxide (H_2_O_2_). DOPAL, in turn, is degraded to 3,4-dihydroxy-phenylacetic acid (DOPAC) catalyzed by the enzyme aldehyde dehydrogenase (ADH). In the presence of ferrous (Fe^2+^), H_2_O_2_ produces free radicals and ROS through the Fenton reaction. COMT converts DA to 3-methoxytyramine, which is then reduced to homovanillic acid (HVA) by MAO (Fig. 1). It is worth noting that COMT is predominantly expressed in glial cells but at very low levels in neurons. MAO-A predominates in catecholaminergic neurons while MAO-B locates mainly in astrocytes [30].

**Fig. 2:**
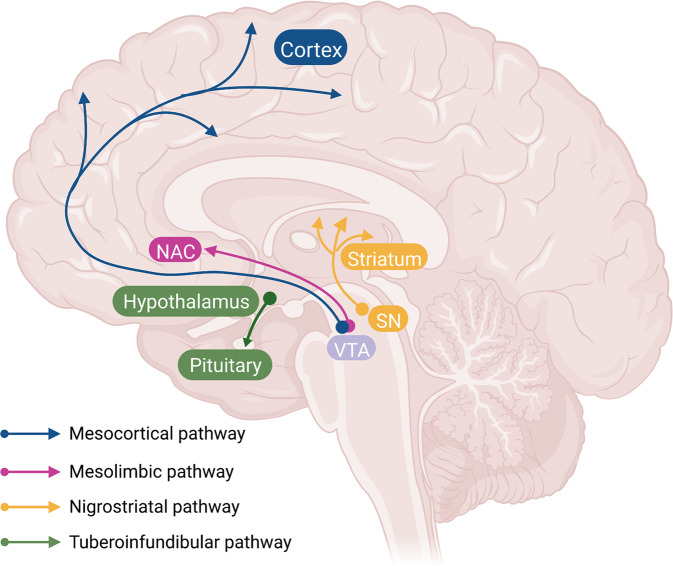
Dopaminergic pathways in the brain. Dopaminergic pathways in the brain include the mesocortical pathway (blue) from dopaminergic neurons in ventral tegmental area (VTA) to cortex, the mesolimbic pathway (red) from VTA to nucleus accumbens, the nigrostriatal pathway (yellow) from substantia nigra to the striatum, and the tuberoinfundibular pathway (green) from hypothalamic nuclei (arcuate nucleus and periventricular nucleus) to the pituitary. The fig was modified from that by Klein et al. (2019)^11^.

Dopaminergic neurons locate principally in the ventral tegmental area (VTA), the substantia nigra pars compact (SNc) of the midbrain, and the arcuate nucleus of hypothalamus. These DA neurons send long-range projections to many sites in the diencephalon and telencephalon. Specifically, the SNc DA neurons project primarily to the dorsal striatum (the caudate/putamen) thus form the nigrostriatal pathway, which has a role in the control of motor function and learning capabilities [31]. The dopaminergic neurons in VTA project to the prefrontal cortex (PFC) via the mesocortical pathway and to the nucleus accumbens via the mesolimbic pathway [32, 33]. Collectively, these pathways form the mesocorticolimbic system, which plays a role in reward and motivation [34]. The fourth pathway is the so-called tuberoinfundibular dopaminergic pathway consisting of the projections from the arcuate nucleus and the periventricular nucleus of the hypothalamus to the pituitary gland and regulating the secretion of prolactin from the anterior pituitary gland [35, 36] (Fig. 2).

The role of DA neurons in motor function can be inferred from patients with PD, a neurodegenerative disease with the degeneration of DA neurons in SNc. PD patients show characterized clinical symptoms of tremor, bradykinesia and rigidity, suggesting a correlation between DA neurons and motor function in humans. Also, this correlation has been demonstrated in animal studies. For example, depletion of striatal DA via the neurotoxin 6-hydroxy-dopamine in rats produces aphagia and akinesia. Furthermore, DA agonists were shown to ameliorate some difficulties in motor initiation and performance in patients and experimental animals [37, 38].

Relevant to but different from the akinetic effects of DA loss in advanced PD and neurotoxin-exposed rats, neither rat nor human PD cases display a fundamental inability to move. Instead, DA-lesioned rats swim in cold water [39] and consume food placed in their mouths while showing signs of enjoying it [40]. Similarly, akinetic patients may get up and run if a fire alarm sounds. However, they will not choose to exert effort to actively obtain rewards [3]. These and many other similar studies established a fundamental link between DA and motivation. Following the same logic, the movement slowing observed in less-severe cases of PD can be considered a motivational deficit [41].

Emerging evidence has demonstrated that DA neurons come in various types that send distinct motivational messages about rewarding and non-rewarding events [42]. It is now known that DA neurons transmit multiple signals resulted from distinct neural processes. Some of the signals reflect detailed predictions about rewarding and aversive experiences, while others indicate fast responses to events of high potential importance. Specifically, some DA neurons support brain systems that assign motivational value, others support neuronal systems engaging in motivational salience [43].

The diverse sub-populations of VTA neurons in mesocortical DA system have distinct roles in aversion, reward, motivation and learning [44]. Moreover, recent studies described new populations of VTA neurons, i.e., VTA-GABA neurons [44, 45] and VTA-glutamate neurons [46]. Both groups of the neurons have been implicated in reward and aversion processes. Specifically, VTA-GABA neurons increase their firing rate when animals are exposed to cues that predict reward and show a transient increase in response to aversive stimuli [47]. Optogenetic activation of VTA-glutamatergic neurons that project to the NAc leads to aversion by activating GABA interneurons, which in turn release GABA onto medium spiny neurons [47, 48]. Taken together, these studies indicate that VTA-glutamate and VTA-GABA neurons interact with each other in regulating multiple behavioral responses.

The mPFC and striatal neurons in the mesocorticolimbic system involve in reward-based associative learning as the blockade of both D1R and D2R in the monkey’s PFC results in learning deficits [49, 50]. Also, D1R antagonism was shown to impair learning on a spatial food-rewarded task whereas D1R agonist infusion resulted in a better performance of the same task [51]. Moreover, hippocampal DA also regulates appetitive memory formation as evidenced by the finding that the D1R blockade prior to a cocaine-conditioning session in a conditioned place preference task impairs short and long-term memory formation [52]. Taken together, these studies demonstrated the modulation of appetitive memories by dopaminergic transmission in the mesocorticolimbic system.

The DA neurons in the hypothalamic arcuate nucleus release DA into hypothalamohypophyseal blood vessels of the median eminence. Through this pathway, DA inhibits the secretion of prolactin from lactotrope cells in the anterior pituitary gland. In contrast, lactotrope cells secrete prolactin continuously in the absence of DA. Therefore, DA is also referred to as the prolactin-inhibiting factor, prolactin-inhibiting hormone, or prolactostatin [53]. In contrast to this prolactin-inhibiting action of DA, antipsychotic drugs given to women lead to hyperprolactinemia with the consequences of amenorrhea, cessation of the normal ovarian cycle, loss of libido, false-positive pregnancy tests, and the long-term risk of osteoporosis. In males, antipsychotics-induced hyperprolactinemia may result in gynecomastia, lactation, impotence, and loss of libido [54].

## Dopaminergic dysfunctions in schizophrenia

Since the dopaminergic system is an important player in multiple functions of human body as reviewed above, it is not surprising that malfunctions of dopaminergic signaling have been implicated in the pathogenesis of various human disorders including PD, HD, ADHD, schizophrenia, and addiction [11]. This article, however, focuses on three lines of evidence for dopaminergic dysfunction in schizophrenia as follows.

First, the clinical effectiveness of antipsychotic drugs is directly related to their affinity for DA receptors [55]. Generally, more than 60% occupancy of D2R is required for a high likelihood of response [56]. This focus on D2R, however, was brought into question by the clinical observations that clozapine is superior to other antipsychotics in antipsychotic-resistant patients despite its rather low affinity for and occupancy at D2R. Moreover, DA metabolite measures were reduced in some patients with schizophrenia [57]. In attempting to reconcile these inconsistencies, the one-sided DA hypothesis was modified into a new version consisting of a prefrontal hypodopaminergia and a subcortical hyperdopaminergia [57, 58]. Supporting evidence for the prefrontal hypodopaminergia came from PET (positron emission tomography) studies showing reduced cerebral blood flow in frontal cortex, which was directly correlated with low cerebrospinal fluid (CSF) DA metabolite levels in schizophrenia patients. Furthermore, animal studies provided direct evidence linking prefrontal hypodopaminergia and subcortical hyperdopaminergia. Specifically, lesions of DA neurons in PFC result in increased levels of DA and its metabolites and D2-receptor density in the striatum [59], whereas the application of DA agonists to PFC reduced DA metabolite levels in the striatum [60].

Secondly, studies with schizophrenia patients have reported elevated presynaptic striatal DA synthesis capacity [61]. Of the schizophrenia patients in the above studies, those who were acutely psychotic at the time of PET scanning showed elevated presynaptic striatal DA availability. Striatal synaptic DA release increased in schizophrenia patients measured by PET and single photon emission computerized tomography (SPECT) following a challenge that releases DA from the neuron [62]. Relevantly, there is a modest elevation in striatal D2/3 receptor density in schizophrenia independent of the effects of antipsychotic drugs [63].

DA abnormalities may be seen in persons prior to the onset of psychosis thus are unlikely a consequence of psychotic episodes or antipsychotic exposure [64]. Furthermore, ultra-high risk (UHR) subjects show elevated subcortical synaptic DA content [65] and basal DA synthesis capacity [66]. Importantly, alterations in DA synthesis capacity in UHR subjects progress over time and are greater in subjects who transition to psychosis relative to those who do not [67].

Different from striatum, dopaminergic transmission in PFC is mainly mediated by D1R. An early study with schizophrenia patients reported decreased prefrontal D1R compared with healthy control subjects revealed by PET [68]. In a small sample of twin pairs discordant for schizophrenia, lower D1R binding was seen in chronic, medicated schizophrenia probands compared with controls [69]. In a recent study, neuroleptic-naive patients showed lower prefrontal D1R availability compared to healthy control subjects revealed by PET, suggesting a reduction of prefrontal D1R density in the pathophysiology of schizophrenia [70]. It is worth noting that the relationship between cortical and striatal DA is bidirectional as evidenced by the finding that increased dorsal striatal dopaminergic signaling led to a reduction in mesocortical DA release while resulted in cognitive deficits [71, 72].

Thirdly, amphetamines and other drugs that release DA induce psychotic symptoms in healthy volunteers and worsen symptoms in patients with schizophrenia [62]. Compared with control participants, patients with schizophrenia showed greater release of DA after amphetamine administration and this increased DA release was directly associated with the worsening of psychotic symptoms in the patients [73]. With the same clinical significance, increased subcortical DA synthesis and release capacity are strongly associated with positive symptoms in schizophrenia patients, and increased subcortical synaptic DA content is predictive of a positive treatment response [74].

Finally, most animal models of schizophrenia report increased locomotor activation following administration of psychostimulants [75]. Indeed, the increased locomotor response to amphetamine and other psychostimulants has been used as a simple test to reflect the subcortical hyperdopaminergia underlying the psychotic symptoms in schizophrenia. Moreover, animal studies reported that pre- and perinatal factors led to long-term overactivity in mesostriatal dopaminergic function [76, 77]. Also, neonatal exposure to toxins resulted in increased DA-mediated behavioral responses [78] and elevated striatal DA release [79]. Similarly, prenatal and neonatal stress, such as maternal separation, promoted striatal DA metabolism [80] and release [81, 82].

## Mitochondria: structure, function, and dynamics

The mitochondrion is a double membrane organelle present in almost all cells. The fine structure of a mitochondrion consists of outer membrane (OM), inner membrane (IM), inter-membrane space between OM and IM, as well as mitochondrial matrix (MM). OM is smooth and serves as boundary membrane of the organelle. IM is highly structured and differentiated into compositionally and functionally distinct regions of the inner boundary region and crista junctions, which are tubules connecting the cristae to the boundary and segregating soluble inter-membrane space from the boundary regions [83].

**Fig. 3:**
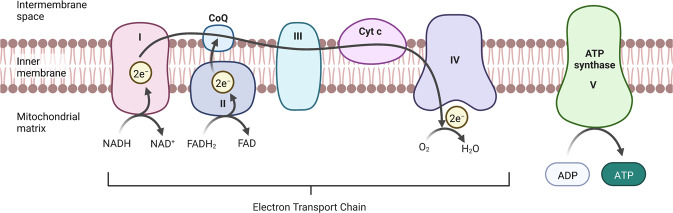
The oxidative phosphorylation complexes in the electron transport chain (ETC) of mitochondrion. Complex I (Co-I) receives electrons from NADH. Complex II (Co-II) represents a point of intersection between tricarboxylic acid cycle and electron transport. Mobile electron carriers, like coenzyme Q (CoQ) and cytochrome c, move electrons between protein complexes. Complex III (Co-III) is the CoQ-cytochrome c oxidoreductase. Complex IV (Co-IV) is also known as cytochrome c oxidase (COX). This complex terminates the flow of electrons through the ETC, reducing oxygen to water. Finally, complex V (Co-V) generates ATP from the energy stored by the proton gradient established through the previous step by step work.

In the mitochondrial matrix, tricarboxylic acid cycle (TCA) enzymes generate electron carriers such as NADH and FADH2, which donate electrons to the electron transport chain (ETC) in the IM. The TCA cycle is composed of a series of eight enzymatic steps that consumes, and then regenerates, citrate. In doing so, it links the metabolism of carbohydrates, fats, and proteins, by acetyl CoA produced from the catabolism of these compounds. After entering TCA cycle, acetyl CoA is oxidized into the reducing agents NADH and FADH. The ETC consists of four oxidative phosphorylation (OXPHOS) complexes (I–IV). The individual redox active complexes shuttle electrons to their final acceptor, i.e., oxygen, and form water. Complex I (Co-I) receives electrons from NADH. Complex II (Co-II) is thought to exist as a separate entity and represents a point of intersection between TCA cycle and electron transport. Mobile electron carriers, like coenzyme Q (CoQ) and cytochrome c, move electrons between protein complexes. Complex III (Co-III) is the CoQ-cytochrome c oxidoreductase, within which the two hemes of cytochrome b and the heme of cytochrome c1 are involved in electron transfer during the Q cycle [84]. Complex IV (Co-IV) is also known as cytochrome c oxidase (COX). This complex terminates the flow of electrons through the ETC, reducing oxygen to water. Finally, complex V (Co-V) generates ATP from the energy stored by the proton gradient established through the previous step by step work [85] (Fig. 3).

In addition to energy production, mitochondria perform multiple other functions including Ca^2+^ homeostasis [86, 87], generation of ROS [88], regulation of apoptosis [89], activation of endoplasmic reticulum-stress response [90], as well as other far-ranging sequelae of mitochondrial dysfunction [91].

Different from the other organelle, mitochondrion contains its own DNA (mtDNA). This circular genome is organized into discrete nucleoids in MM. The human mtDNA consists of 16,569 base pairs that encodes for 13 essential polypeptides of the mitochondrial respiratory chain (MRC), two ribosomal RNAs, 22 specific transfer RNAs, and a displacement loop [92]. The 13 mtDNA-encoded polypeptides take a small proportion of the total 90 MRC polypeptides, but they are vital to proper function of MRC [93].

Interestingly, the closest relatives of many mtDNA-modifying enzymes, such as mtDNA polymerase, are bacteriophage proteins [94], suggesting that an infection of the mitochondrial ancestor contributed to the development of mtDNA maintenance machinery. Indeed, there is a theory proposing that mitochondrial ancestor is the bacteria that invaded into archaea [95]. During the evolution process from prokaryotes into eukaryotes, the bacteria became mitochondria and shed most of their genetic material [96]. Different from prokaryotes, which are sexless and reproduce by making copies of themselves, eukaryotes reproduce sexually [97]. The sexual reproduction passes on not only genes but also all the rest of the fertilized egg, including living mitochondria. Importantly, eukaryotic parents avoid mixing their mitochondria while passing their genes; only the mother passes them on [98]. In line with this theory, human mtDNA, unlike nuclear DNA, is passed on only from mothers [99].

It is noteworthy that mitochondria are highly dynamic organelles. They continuously change their function, position, and structure to meet the energetic demands of a given cell [100, 101]. This is not surprising given that the presence of complexes I–V, and their proportion relative to the amount of inter-membrane space, MM, and mitochondrial size directly influences the amount of ATP produced from a mitochondrion [102]. Moreover, mitochondria can modulate their morphology and regulate their number, size and position within the cytoplasm by two opposite processes, i.e., mitochondrial fission and fusion [103]. Mitochondrial fission is a multi-step process that allows the division of one mitochondrion into two daughter mitochondria thus contributing to a chance for a mitochondrion to fuse with another part of the mitochondrial network [104, 105]. Mitochondrial fusion is a two-step process starting from OM fusion and ended with IM fusion. This process promotes complementation between two mitochondria, including mtDNA [106, 107].

A place where mitochondrial trouble occurs frequently is the brain [108]. Indeed, mitochondrial defects have been linked to some of neurodegenerative diseases. For example, poor transport of mitochondria precedes the onset of AD and PD [109]. Moreover, mitochondrial defect is an important component in the pathogenesis of schizophrenia as reviewed in the next section.

## Mitochondrial defects in schizophrenia

Given its multiple functions in neurons and glial cells, mitochondrial malfunctioning has been associated with virtually every mental or neurological affliction in humans, including chronic psychological stress and fatigue, cognitive deficits, Alzheimer’s disease (AD), PD, anxiety, depression, bipolar disorder, schizophrenia, autism, multiple sclerosis, and Down syndrome. Here we reviewed only evidence for mitochondrial defects in schizophrenia.

Earlier epidemiological data support the existence of pathogenic genes located in mtDNA that may persist through the matrilineal inheritance mechanism in schizophrenia [110]. In the same line, a later cross-sectional study found that risk for schizophrenia has a maternal inheritance bias [111]. In line with these earlier studies, a recent study with Han Chinese showed maternal inheritance of mtDNA variants in schizophrenia as evidenced by sequencing data of the entire mitochondrial genomes of probands from 11 families with a family history and maternal inheritance pattern of schizophrenia [112].

A study with a large sample set identified a large number of significantly altered genes encoding mitochondrial and mitochondria-related proteins. Of these genes, significantly down-regulated ones are those for oxidative phosphorylation, energy pathways, RNA metabolism, vesicle transport, protein transport, carbohydrate biosynthesis, lipid biosynthesis and glycolysis pathways. Further hierarchical clustering identified 59 genes that are related mainly to mitochondria and energy metabolism [113]. Moreover, the results from the Schizophrenia Psychiatric Genomics Consortium (PGC-SCZ2) GWAS (35,476 cases and 46,839 controls) provided evidence for 22 nuclear-encoded mitochondrial genes [114]. In a more recent study, Gonçalves et al. conducted gene-based and gene-set analyses in which 1186 mitochondrial genes were analyzed. They reported that 159 genes had *p*-values < 0.05 and 19 remained significant after multiple testing correction. Furthermore, they did a meta-analysis of 818 genes combining the PGC-SCZ2 and iPSYCH samples and revealed 104 nominally significant and nine significant genes, confirming the involvement of the nuclear-encoded mitochondrial genes in schizophrenia [115].

A great number of mtDNA single nucleotide polymorphyisms (SNPs) are strongly associated with schizophrenia. Of them, two SNPs in the gene encoding NADH dehydrogenase of Co-I are positively correlated with schizophrenia [116, 117]. Moreover, several SNPs in the gene encoding ATP synthase subunit six are associated with increased risk of developing schizophrenia [118]. In a recent study with Han Chinese, expression of 13 mitochondrial (MitoCarta) genes were found to be significantly decreased in hippocampal neurons of patients with schizophrenia as compared to control subjects [119]. According to a comprehensive review, 295 mitochondrial genes were identified to be associated with schizophrenia in at least 1 study. Fifty-seven of these mitochondrial genes were associated with schizophrenia etiology or pathology in at least 2 independent studies [120]. The 57 mitochondrial genes are associated with protein products shown by synaptic and non-synaptic enrichment. The proteins with synaptic enrichment involve in ATP metabolism and the ETC, while proteins with non-synaptic enrichment involve in TAC, protein transport and folding, as well as the ETC [121]. Relevantly, the protein product of 42 mitochondrial genes was altered in brains of patients with schizophrenia. Notably, 18 of these 42 mitochondrial genes were found to be independently associated with schizophrenia [122]. Moreover, mutations in a gene encoding a tRNA [Leu(UUR)] is associated with schizophrenia [123].

Several copy number variant (CNV) in mitochondrial genes have been implicated in schizophrenia etiology [124]. A known example is the genome-wide CNV analysis of the Swedish cohort, which reported a significant enrichment of the mitochondrial network in schizophrenia, using both a smaller reference list of 193 genes and a larger one of 892 mitochondrial genes representative of the majority of validated murine mitochondrial genes in MitoCarta [125]. In addition, nuclear-encoded mitochondrial genes are also implicated in schizophrenia etiology and pathology as evidenced by the 22q11.2 deletion syndrome resulted from a micro-deletion in human chromosome 22. Up to one third of individuals with 22q11.2 deletion syndrome develop schizophrenia or schizo-affective disorder by adolescence or early adulthood [126]. Another example is DISC1 (disrupted in schizophrenia 1), which is perhaps the best characterized generalized risk factor for major psychiatric disorders. It was first identified in cytogenetic screens of a large Scottish family with high prevalence of schizophrenia and related psychoses [127]. Two splice variants of DISC1 have been localized to mitochondria and mutations in DISC1 have also been linked to altered mitochondrial dynamics [128, 129]. Furthermore, schizophrenia-associated DISC1 fusion and truncation mutant proteins were shown to inhibit mitochondrial trafficking and fusion thus disrupted normal dendritic development of cultured neurons [130, 131].

The second line of evidence for mitochondrial defects is changes in mitochondrial morphology, density and number seen in brains of patients with schizophrenia. In an early study, patients with schizophrenia showed a significant reduction in mitochondrial cross-sectional profiles in the caudate and putamen [132]. Importantly, a reduction in mitochondrial density was observed in oligodendrocytes of brains of schizophrenia patients [133]. Moreover, schizophrenia patients showed 26–30% fewer mitochondria per synapse in stratum compared to controls [134]. Similarly, fewer mitochondria were seen in lymphocytes from drug-free schizophrenia patients [135, 136]. In addition, mononuclear cells of schizophrenia patients showed enlarged mitochondria with fragmented cristae, which was independent of antipsychotic drug used [135]. Importantly, long duration of schizophrenia was found to be associated with enlarged mitochondria with destroyed cristae in astrocytes [137].

In addition to the electron and fluorescence microscopy analyses of post-mortem brain tissue from schizophrenia patients, functional signatures of mitochondrial dysfunction have been observed in induced pluripotent stem cell (iPSC) models derived from schizophrenia patients. For example, abnormal neuronal differentiation and mitochondrial malfunction were seen in hair follicle-derived iPSC of schizophrenia patients [138]. In a recent study, iPSCs from schizophrenia patients showed abnormal gene expression and protein levels related to cytoskeletal remodeling and OS [139]. In a more recent study with iPSC-derived cerebral organoids from patients with schizophrenia, RNA sequencing data showed differential expression of genes involved in synapses, neural development, and antigen processing. Analysis of the gene expression profiles suggested dysregulation of genes involved in mitochondrial function or in modulation of excitatory and inhibitory pathways [140].

Another line of evidence for mitochondrial defects in schizophrenia is mitochondrial OS demonstrated in many clinical studies. A meta-analysis of 44 studies reporting OS markers in serum, plasma, and erythrocytes in patients with schizophrenia or related psychotic disorders concluded that the changes in specific parameters were correlated with the clinical status of schizophrenia [141]. Moreover, a recent review listed the 100 studies that identified an association between OS and schizophrenia [142]. Relevantly, several studies reported lower glutathione (GSH) levels in peripheral samples, CSF, and post-mortem caudate nucleus and PFC from drug-naive or treated patients compared with controls [143]. In addition, the brain of schizophrenic patients contains increased levels of oxidative damage products such as 4-hydroxy-2-nonenal, confirming the presence of OS [144]. Importantly, serum OS determinations correlate with the occurrence and course of schizophrenia [145].

More direct evidence for mitochondrial defects in schizophrenia came from noninvasive neuroimaging studies using phosphorous or hydrogen magnetic resonance spectroscopy (^31^P-MRS and ^1^H-MRS, respectively). Earlier studies in this line revealed reduced ATP and phosphocreatine (PCr) in the frontal lobe, the caudate nucleus and the left temporal lobe of schizophrenic patients [146, 147]. A later study with schizophrenia showed decreased ATP production but increased anaerobic metabolism of glucose. Relevantly, excessive lactate production was observed in CSF of the patients [148]. A systemic review, however, concluded with no consistent patterns for the comparison of energy related phosphorus metabolites between schizophrenia patients and controls. The authors speculated that methodological heterogeneities and shortcomings in the literature likely obscured consistent patterns among studies. And recommended to improve study designs and ^31^P-MRS methods in future studies [149].

Last but not least, schizophrenia or psychiatric symptoms are also seen in mitochondria diseases. In this aspect, Anglin et al. described a series of 12 patients with mitochondrial disorders in whom psychiatric symptoms were a prominent aspect of the clinical presentation [150]. The same group also searched the literature and found fifty cases of mitochondrial disorders with prominent psychiatric symptomatology. In the cases of mitochondrial disorders, the most common psychiatric presentations were mood disorder, cognitive deterioration, psychosis, and anxiety. The most common diagnosis (52% of cases) was a MELAS (mitochondrial encephalomyopathy with lactic acidosis and stroke-like episodes) mutation [151].

## Interplay of dopamine metabolism abnormalities and mitochondrial defects

Under physiological condition, DA in synaptic vesicles is stabilized by the slightly acidic pH there [25]. This optimal microenvironment, plus the tight associations of the enzymes TH and AADC with VMAT2, prevent the autoxidation of DA in the cytosol thereby avoiding cellular OS [152]. Nevertheless, DA may accumulate in the cytosol as a consequence of leakage from synaptic vesicles. The accumulated DA is then degraded through the oxidative deamination reactions catalyzed by MAO and COMT producing H_2_O_2_, in addition to its metabolites [153].

DA in cytosol can be taken up by the mitochondria therein it reversibly inhibits NADH (Co-I) activity in ETC of mitochondria as seen in rat brain [154]. Also this toxic effect of DA on mitochondrion has been demonstrated in cultured SH-SY5Y cells where DA significantly dissipated mitochondrial membrane potential (∆Ψ) while inhibited Co-I activity. Moreover, increased susceptibility of Co-I activity to DA inhibition was seen in platelets of schizophrenic patients, suggesting a pre-existing deficient Co-I in mitochondria of the patients [155]. Indeed, abnormalities have been observed in Co-I subunits located at the suggested interaction site of Co-I with DA [156]. Interestingly, DA inhibited mitochondrial function in a dose-dependent manner as evidenced by DA-induced decrease in intracellular ATP, ∆Ψ, Co-I activity, and cell viability, while the treatment increased intracellular ROS level in cultured mature oligodendrocytes (OLs). Moreover, these effects were effectively ameliorated in the presence of tranylcypromine (a non-selective and irreversible MAO inhibitor), suggesting that the toxic effects were due to ROS resulted from the oxidative deamination reactions of DA, but not DA per se; i.e., it is the ROS that damaged mitochondria in the cells (unpublished data).

It is worth noting that DA is easily oxidizable. Specifically, superoxide anion promptly accepts an electron from DA, which is transformed into the o-semiquinone radical. Then two such radicals disproportionate thus generating a dopaminoquinone (DA-Q) plus a DA. The unstable DA-Q undergoes spontaneous 1,4-intramolecular cyclization and further oxidation, eventually forming the relatively stable dopaminochrome (DAC), a 5-dihydroxyindole tautomer, which is cytotoxic in an OS-dependent manner [157, 158].

Under physiological conditions, the rate of DA oxidation is slow, and the cellular antioxidant machinery can cope with the formation of highly reactive products from DA oxidation [13]. In a state of increased oxidation, however, the cellular antioxidative machinery fails thus leading to massive oxidation of DA, which damages essential cellular function even results in cell death. Indeed, there are a wide range of experimental data demonstrating toxic effects of higher levels of DA on mitochondria of neurons and glia cells. An example of early studies is the DA-induced apoptosis in a cultured neuronal model due to its toxic effects on mitochondria [14]. This mitochondrial defect mechanism was further demonstrated in the second study by the same group as the DA-induced apoptosis in mesencephalic cells was enhanced by cyanide, which is toxic to mitochondrion and stimulates intracellular generation of ROS [159]. Studies by the other groups showed that oxidized DA is cytotoxic to both MN9D (a mesencephalic cell line) and PC12 cell lines in a manner of stimulating OS [160, 161]. Also, DA triggers apoptosis in pituitary cells via a mechanism involving OS as evidenced by DA-induced loss of ∆Ψ, relocation of Bax to the mitochondria, cytochrome c release, caspase-3 activation, and nuclear fragmentation [162]. In addition, it was demonstrated that DAC causes MN9D cell death in a caspase-independent apoptotic manner that involves oxidative damage to DNA [158]. The toxic effects of DA oxidation were also seen in astrocytes as shown in a recent study showing that DOPAL significantly reduced Neu7 (a rat astrocyte cell line) viability, induced apoptosis, decreased mitochondrial performance, and increased oxidative and nitrative stress, in a dose-dependent manner [163].

The other side of interaction between DA and mitochondria is the effect of deficient mitochondrion on DA catabolism. In this regard, the author’s group did a series of work with the cuprizone-exposed mouse as an animal model of schizophrenia to explore the interaction between abnormal dopamine metabolism and mitochondria defects in brain cells. Cuprizone is a chemical chelator toxic to mitochondria of cells [164]. In addition to suppressing activities of Co I–IV in MRC of mitochondrion [165], cuprizone also inhibits copper-dependent enzymes such as cytochrome c oxidase, dopamine β-hydroxylase (DBH), and SOD thereby increasing the production of ROS in the cytosol [166].

As expected, cuprizone-feeding impaired mitochondrial function of brain cells of living C57BL/6 mice as evidenced by decreased levels of N-acetyl-L-aspartate (NAA), total NAA (NAA and NAAG), and choline-containing compounds (phosphorylcholine and glycerophosphorylcholine) detected by ^1^H-MRS, in addition to inducing demyelination and OL loss seen in post-mortem brain tissue. The treatment also decreased activities of catalase and glutathione peroxidase, but increased levels of malondialdehyde and H_2_O_2_ in the brain tissue, indicating the presence of OS there [167]. Moreover, cuprizone-feeding induced a transitional changes in DA and NE levels in PFC of the mouse (higher DA and lower NE) while decreased activities of MAO and DBH in the hippocampus and PFC of the subjects as compared to normal controls. Interestingly, the cuprizone-fed mice showed a couple of behavioral abnormalities including increases in climbing behavior and time remaining in open arms of the elevated plus maze, impaired prepulse inhibition (PPI) and spatial working memory, as well as decreased social interactive behaviors. These behavioral changes are reminiscent of some clinical manifestations seen in schizophrenia patients [15]. Following our studies, the other investigators replicated the same findings seen in cuprizone-exposed mice and rats [168–170]. Taken together, the previous studies have demonstrated that functional impairment of mitochondrion may impair DA catabolism thus leading to elevated levels of DA and ROS in brain cells.

In support of the interaction between mitochondrial defects and abnormal DA metabolism in the pathogenesis of schizophrenia, antipsychotic drugs protected against the cuprizone-induced changes or promoted the recovery processes of the changes in mice. Specifically, haloperidol improved PPI deficit, clozapine and quetiapine improved more behavioral abnormalities while ameliorated the cuprizone-induced white matter damage [171–173]. The additional therapeutic effects of clozapine and quetiapine were attributed to the antioxidant and anti-inflammatory actions of the atypical antipsychotics as demonstrated in animal and cell culture studies [167, 170, 174–177]. In line with these preclinical studies, clinical practice also reported preferable outcomes of negative symptoms and cognitive impairment in some patients with schizophrenia treated with atypical antipsychotics compared to those treated with typical ones [178, 179]. Indeed, in the majority of schizophrenia patients, administration of atypical antipsychotics seemed to result in higher levels of patient satisfaction than did conventional drugs [180].

The interaction between DA and mitochondrion in brain cells was also demonstrated in the studies with rotenone, a specific inhibitor of mitochondrial Co-I. An acute exposure to rotenone was reported to result in ubiquitin and α-synuclein-positive inclusions and led to selective dopaminergic neuronal damage due to the inhibition of mitochondrial Co-I [181]. Moreover, a single systemic rotenone administration to rats did not lead to neurotoxicity, but rather to enhanced glutamate-induced DA release in brain cells [182], suggesting a possibility that DA catabolism was inhibited due to functional impairment of mitochondrion by rotenone in the cells. In line with this previous study, oral medication of rotenone to mice increased DA level in PFC, decreased production of ATP in PFC, hippocampus, and caudate putamen, and led to impairment in learning and executive function of the subjects (unpublished data).

## Antioxidant addition to antipsychotic treatment for schizophrenia

Along with the studies showing evidence for OS in schizophrenia patients, there is increasing clinical studies applying antioxidant addition to antipsychotic treatment for patients with schizophrenia. The antioxidants commonly added as adjunctive medication for patients with schizophrenia include vitamin C and E, N-acetylcysteine (NAC), ginkgo biloba, minocycline, resveratrol, and omega-3 fatty acids. Because of the space limitation, the following paragraphs just outline some of clinical reports/studies using antioxidant addition to antipsychotic treatment for schizophrenia, instead of providing a complete review of all the extant studies.

As early as in 1963, Milner reported a double-blind trial of ascorbic acid (vitamin C) in 20 male chronic psychiatric patients [183]. Administration of vitamin C (1 g/day) for three weeks resulted in a significant improvement in the depressive, manic, and paranoid symptom complexes in the patients. Later on, Beauclair and colleagues reported an open label trial with schizophrenic patients on stable unspecified neuroleptic regimens. Vitamin C administration for a period of 8 weeks resulted in symptomatic improvement in 10 of 13 patients [184]. In a pilot study by Kanofsky et al., 21 refractory schizophrenia inpatients received ascorbate (2–6 g/day) adjunctively with their ongoing neuroleptic medication for a minimum of one month. Seven of these patients showed definite clinical improvement [185]. In a case report by Sandyk and Kanofsky, a 37-year-old chronic schizophrenia patient derived substantial benefit from the addition of vitamin C to his neuroleptic treatment [186]. Moreover, oral supplementation of vitamin C with atypical antipsychotic were shown to reverse ascorbic acid levels, reduce OS, and improve BPRS (brief psychiatric rating scale) score in forty schizophrenic patients participated in a prospective, double-blind, placebo-controlled, 8-week study [187]. Nevertheless, we noticed a report stating that the addition of vitamin C was not associated with any change in psychopathology in 8 male inpatients diagnosed as chronic schizophrenia in a study by Straw and colleagues [188].

In 2008, Berk et al. conducted a randomized, multicenter, double-blind, placebo-controlled study aiming to evaluate the safety and effectiveness of oral NAC as an add-on to maintenance medication for chronic schizophrenia over a 24-week period. They reported a greater improvement in patients treated with NAC than placebo-treated subjects over the study period in terms of PANSS (Positive and Negative Symptoms Scale) total, PANSS negative, and PANSS general, CGI- (Clinical Global Impression) Severity (CGI-S), and CGI- Improvement (CGI-I) scores [189]. These results suggest that adjunctive NAC has potential as a safe and moderately effective augmentation strategy for chronic schizophrenia. In a relatively small sized, randomized, double-blind, placebo-controlled study with 42 chronic schizophrenia patients, NAC-treated subjects showed significantly greater improvement in the PANSS total and negative subscale scores compared to the placebo group, but there was no difference between the 2 groups in the frequency of adverse effects [190]. Similarly, a 12-week, double-blind, randomized, placebo-controlled, clinical trial reported improvement in positive, negative, general and total psychopathology symptoms as well as cognitive performance in NAC group over placebo group. NAC was also well-tolerated, safe and easy-to-use as an effective therapeutic strategy to improve outcome in patients with schizophrenia [191].

Some but not all the previous findings of NAC efficacy were replicated in a recent 52-week, double-blind, placebo-controlled trial on symptoms and cognition in early phase schizophrenia spectrum disorders. Specifically, NAC significantly improved PANSS total, negative, and disorganized thought symptom scores, but failed to improve PANSS-positive symptoms and BACS (Brief Assessment pf Cognition in Schizophrenia) cognitive scores [192]. In another study published in the same year, however, NAC supplementation in a limited sample of early psychosis patients did not improve negative symptoms while led to some neurocognitive improvements [193]. In a recent clinical trial with a sample of 58 participants randomized in a double fashion to receive NAC or placebo for 24 weeks, patients treated with NAC had significantly higher working memory performance at week 24 compared with placebo, suggesting that NAC has an impact on cognitive performance in psychosis [194]. The above inconsistency between individual clinical studies has been reviewed and analyzed in a more recent meta-analysis of randomized controlled trials with NAC in the treatment of schizophrenia patients from seven studies including 220 receiving NAC and equal number of 220 receiving placebo. It concluded that NAC significantly improved PANSS negative and total scores, as well as improved the cognitive domain of working memory after 24 weeks of treatment [195].

In addition to vitamin C and NAC that have been used as antioxidant addition to antipsychotic treatment for schizophrenia, the others that have been tested in clinical trials with schizophrenia patients include ginkgo biloba [196, 197], allopurinol [197], minocycline [198], resveratrol [199], essential polyunsaturated fatty acids (EPUFAs), particularly, omega-3 fatty acids [200], vitamin E [201], as well as a mixture of omega-3 and vitamin E/C [202]. Overall, there is preliminary data that suggests substances with antioxidant potential may be of use, although definitive studies are needed. Specifically, ginkgo and NAC emerged as the most promising antioxidants thus may be added to antipsychotic treatment for schizophrenia patients.

## Concluding remarks

In summary, schizophrenia is a genetically and phenotypically complex brain disease, driven by a combination of genomic and environmental factors [203]. Of the genetic factors, 18 of the 42 mitochondrial genes have been found to be independently associated with schizophrenia [122], in addition to those nuclear-encoded mitochondrial genes implicated in schizophrenia etiology and pathology as evidenced by the 22q11.2 deletion syndrome [126]. As a result of these genetic changes, mitochondrial abnormalities in either density, morphology, and/or function occur. Environmental factors, including pre- and perinatal factors leading to long-term overactivity in mesostriatal dopaminergic function, are also able to inhibit mitochondrial function. Under both the genetic and environmental conditions resulting in mitochondrial defects, DA catabolism in mitochondrion is inhibited along with increased ROS but decreased ATP in the organelle. On the other hand, DA increase, which may be due to inhibition of its catabolism enzymes such as COMT or MAO by genetic deletion and pharmacological approaches, would impair mitochondrial function as evidenced by the inhibition of Co-I activity in MRC. It is the vicious cycle from mitochondrial defects to DA increase or vice versa that play critical roles in the pathogenesis of schizophrenia.

In addition to contributing to positive symptoms in schizophrenia, elevated DA and mitochondrial defects may result in neurodevelopmental abnormalities including reduced spine density in cortical neurons, NMDA-R hypofunction [204], hypomyelination or white matter abnormalities seen in brains of patients with schizophrenia [204, 205]. These neurobiological changes have been thought to account for the negative symptoms and cognitive impairment in schizophrenia [204, 206].

All extant antipsychotics are D2-receptor blockers. Typical antipsychotics exert their therapeutic efficacy on positive symptoms via substantial occupancy (more than 60%) of D2 receptors [56], but greater than 80% occupancy increases the likelihood of movement-associated adverse effects [207]. While having lower D2 occupancy relative to typical antipsychotics, atypical ones are effective on both positive and negative symptoms, as well as cognitive impairment, but less likely to induce movement-associated adverse effects in patients with schizophrenia [206]. These preferable efficacy of atypical antipsychotics on negative symptoms and cognitive impairment in patients with schizophrenia may be related to the antioxidant and anti-inflammatory actions of these drugs as demonstrated in animal and cell culture studies [167–169, 174–177].

Oxidative stress resulted from mitochondrial defects and the concurrent neuroinflammation, two pathological components involved in the pathogenesis of schizophrenia, calls for combination of antipsychotic drug and antioxidants in treating the patients. To date, a lot of antioxidants have been applied in clinical trials with schizophrenia patients. Overall, emerging data are promising at least from some of studies with ginkgo and NAC [197, 201]. More and more clinical trials with antioxidant addition to antipsychotic treatment for patients with schizophrenia are expected.
